# The intestinal B-cell response in celiac disease

**DOI:** 10.3389/fimmu.2012.00313

**Published:** 2012-10-04

**Authors:** Luka Mesin, Ludvig M. Sollid, Roberto Di Niro

**Affiliations:** Centre for Immune Regulation, Department of Immunology, Oslo University Hospital-Rikshospitalet, University of OsloOslo, Norway

**Keywords:** celiac disease, autoantibodies, mucosal immunity, intestinal mucosa, B cells

## Abstract

The function of intestinal immunity is to provide protection toward pathogens while preserving the composition of the microflora and tolerance to orally fed nutrients. This is achieved via a number of tightly regulated mechanisms including production of IgA antibodies by intestinal plasma cells. Celiac disease is a common gut disorder caused by a dysfunctional immune regulation as signified, among other features, by a massive intestinal IgA autoantibody response. Here we review the current knowledge of this B-cell response and how it is induced, and we discuss key questions to be addressed in future research.

The immune system has evolved multiple strategies to maintain intestinal homeostasis. A unique feature of intestinal immunity is the ability to provide protection toward pathogens while preserving the number and composition of the commensal bacteria in a state of mutualism (Hooper and Macpherson, 2010). Secretory IgA is considered to be one of the crucial immune effector mechanisms in maintaining homeostasis at mucosal surfaces (Brandtzaeg et al., 1999; Strugnell and Wijburg, 2010). Therefore, it is not surprising that the mucosal immune compartment is tightly regulated. In this review, we aim at summarizing and discussing the basic concepts of intestinal immunobiology in the context of a prevalent disorder, celiac disease. The study of this condition provides unique understanding of how an intestinal IgA response is induced and reshaped from the healthy to the affected intestinal state, as well as it pinpoints areas of scarce knowledge and poses key questions for future research.

## FEATURES OF INTESTINAL IMMUNITY

### THE INTESTINAL IMMUNE SYSTEM

The generation of secretory IgA is confined to intestinal lymphoid microenvironments that are composed of the inductive and the effector tissue compartments (Brandtzaeg and Pabst, 2004). The inductive compartment of the intestinal immune system consists of gut-associated lymphoid tissue (GALT) and the regional lymph nodes, whereas the effector compartment consists of the lamina propria (LP) and surface epithelia. Together, these form the largest effector organ of humoral immunity, containing at least 80% of the body immunoglobulin (Ig)-producing cells (Brandtzaeg and Johansen, 2005).

### GUT-ASSOCIATED INDUCTIVE LYMPHOID TISSUE

The GALT is the main site for the induction of mucosal IgA B cells. The GALT is comprised of aggregated lymphoid follicles, termed Peyer’s patches and isolated lymphoid follicles. Gut-associated lymphoid follicles are organized structures covered with a specialized follicle-associated epithelium that contains microfold cells (M cells; Neutra, 1999). In the canonical pathway, antigens from the gut lumen are internalized and delivered to subepithelial dendritic cells (DCs) via M cells or receptor-mediated endocytosis by epithelial cells (Neutra et al., 2001). Antigen-loaded DCs migrate from the subepithelial dome into the perifollicular T cell-rich area, where they can induce a response of helper T cells (Rimoldi et al., 2005). B cells become activated by the presentation of antigens from follicular DCs (Harwood and Batista, 2010) and by CD40-mediated signals from antigen-primed helper T cells (Elgueta et al., 2009). Gut-associated lymphoid follicles are, therefore, characterized by germinal centers (GC) that promote antigen-specific interaction between T and B cells, an essential mechanism for B cell differentiation and diversification. The GALT further drives intestinal IgA production by providing cytokines with IgA-inducing functions, including transforming growth factor-β (TGF-β; Gonnella et al., 1998; Stavnezer and Kang, 2009), retinoic acid (RA; Mora et al., 2006), IL-6 (Sato et al., 2003), and inducible nitric oxide synthase (iNOS; Tezuka et al., 2007). These events lead to up-regulation of the gene encoding for the enzyme activation-induced deaminase (AID), which is central to both class-switch recombination and somatic hypermutation of Ig genes (Muramatsu et al., 2000). This T cell-dependent pathway usually results in generation of plasma cells (PCs) producing intestinal IgA antibodies with high rates of somatic hypermutation (SHM), as well as memory B cells (Bemark et al., 2012). CD40-mediated signal from T cells is crucial to GALT GC initiation (Bergqvist et al., 2006), although it is possible that human intestinal GC-associated B cell responses are not exclusively dependent on cognate T cell/B cell interactions (Spencer et al., 2012). Studies have indicated that GCs can appear in Peyer’s patches and mesenteric lymph nodes without the need for the classic T cell/B cell interaction based on BCR specificity (Casola et al., 2004). Accordingly, SHM can take place as an antigen-independent process without being necessarily linked to affinity maturation (Reynaud et al., 1995; Casola et al., 2004). B cells that are activated in this way are thought to depend on “bystander” T cell help in the form of cytokines such as IL-5, IL-6, and IL-10 in order to induce an IgA response (Jiang et al., 2004).

T cell-independent intestinal IgA responses have been reported in both humans (Ferrari et al., 2001; Levesque et al., 2009) and mice (Guy-Grand et al., 1975; Mombaerts et al., 1994). In mice a T cell-independent, antigen-driven pathway in response to highly conserved microbial antigens recognized by Toll-like receptors (TLRs; Cerutti, 2008) generates a primitive intestinal IgA antibody repertoire to commensal bacteria (Macpherson et al., 2000; Bergqvist et al., 2006, 2010). TLR-triggered class switching to IgA is mediated by BAFF and APRIL expression in DCs, monocytes, macrophages, granulocytes, and intestinal epithelial cells (Fayette et al., 1997; Litinskiy et al., 2002; Cerutti et al., 2011). T cell-independent intestinal IgA responses in mice originate from peritoneal and intestinal LP B-1 cells as well as conventional follicular B-2 cells (Fagarasan et al., 2010). Thus, it has been suggested that the intestinal LP may act as a potential site for T cell-independent IgA induction. A lack of consensus, as well as a lack of homology between murine and human models (Gibbons and Spencer, 2011) as the human equivalent of mouse B-1 cells is still not well described (Descatoire et al., 2011), does however exist when it comes to the LP being a site for IgA class switching and diversification (Fagarasan and Honjo, 2000; Macpherson et al., 2000; Fagarasan et al., 2001; Shikina et al., 2004; Boursier et al., 2005; Crouch et al., 2007; He et al., 2007; Barone et al., 2009). Although T cell-independent responses are biologically possible and certainly relevant in mice, in humans their contribution to the intestinal IgA repertoire remains uncertain.

### GUT-ASSOCIATED EFFECTOR LYMPHOID TISSUE

The intestinal LP acts as the effector compartment of the humoral mucosal immune response, and contains predominantly terminally differentiated PCs (Farstad et al., 2000). These are characterized by the expression of the markers CD138 and CD27, whereas levels of CD19 and CD45 are more heterogeneous; the lack of expression of the Ki-67 marker indicates that these cells are not blasting and should therefore be referred to as PCs rather than as plasmablasts (Di Niro et al., 2010). Human intestinal PCs preferentially produce IgA dimers (80%) and IgM pentamers (20%), whereas a small fraction produces IgG (Brandtzaeg et al., 1999; Brandtzaeg and Johansen, 2005). The amount of Ig produced by intestinal PCs is massive, being estimated at 3 g/day in adults (Brandtzaeg and Johansen, 2005). Dimeric IgA is formed of two monomeric IgA units joined by a bridging J chain that is recognized by the polymeric Ig receptor (pIgR), an antibody transporter expressed on the basolateral surface of epithelial cells (Brandtzaeg and Prydz, 1984; Johansen et al., 2001). Upon interaction with pIgR, dimeric IgA is translocated to the surface of epithelial cells as secretory IgA where it exerts its function of immune exclusion, intracellular neutralization, and antigen excretion (Cerutti and Rescigno, 2008; Strugnell and Wijburg, 2010). The intestinal LP in addition to PCs contains a variety of other cell types, including macrophages, DCs, and neutrophils which in association with epithelial cells may play a crucial role in creating and maintaining the niche for PC survival (Benson et al., 2008).

Antigen-specific IgA antibodies generated upon immunization can be identified only locally (Ogra and Karzon, 1969) or both systemically and locally (Crabbe et al., 1969) depending on the immunization strategy. These observations, together with the fact that IgA reactive to commensal bacteria is exclusively present in gut secretions (Macpherson et al., 2000), support the notion of mucosal immunity as an independent compartment. Rotavirus-specific memory B cells were shown to have different antibody repertoires (Weitkamp et al., 2003) than effector mucosal PCs (Di Niro et al., 2010), which raises some questions about the nature of the interplay between these two compartments.

### THE INTESTINAL IgA REPERTOIRE

In principle, both unmutated IgA antibodies with broad reactivity to self and non-self antigens and somatically mutated antigen-specific IgA antibodies could contribute to the intestinal PC repertoire. There are several observations suggesting that the latter pathway may be dominating. Barone et al. (2011) reported results supporting GC origin of human intestinal IgA-producing PCs. Cloning and sequencing of Ig variable region genes of IgA PCs from LP of human small intestine revealed uniformly high degrees of SHM and high ratios of replacement to silent (R/S) mutations in complementarity determining regions (CDR), which argues in favor of antigen-mediated selection (Dunn-Walters et al., 1997; Boursier et al., 1999). Our group isolated human intestinal PCs specific to rotavirus and cloned antibody genes, observing high numbers of mutations (17 mutations per sequence on average, with an R/S of 2.3, in the VH only; Di Niro et al., 2010). Similarly, Benckert et al. (2011) cloned hundreds of human monoclonal antibodies (hmAbs) from IgA and IgG intestinal plasmablasts from the ileum of healthy donors. Regardless of their reactivity, the antibodies had many mutations, averaging 23 in the VH. The majority of the antibodies were specific for foreign or self antigens whereas 25% were polyreactive. Only 7% of IgA displayed cross-reactivity with diverse bacterial strains. To note, the polyreactive antibodies also had high degree of SHM suggesting that antibody polyreactivity of gut plasmablasts may be acquired by somatic mutations. Selection of somatically mutated variants of polyreactive antibodies may simultaneously act as a parallel mechanism in defining and contributing to antigen-specific immune response against foreign antigens. Revision of light chains expressed by IgA PCs is another distinct feature of human intestinal PCs and it is confined to gene rearrangements at the lambda loci (Su et al., 2008). This has been suggested as a beneficial mechanism in order to diversify the intestinal IgA repertoire and to remove non-functional or autoreactive antibodies.

Contrary to initial studies suggesting that the intestinal IgA repertoire is oligoclonal and of low diversity (Dunn-Walters et al., 1997; Holtmeier et al., 2000; Yuvaraj et al., 2009), Lindner et al. (2012) using a high-throughput sequencing method, recently demonstrated that the intestinal IgA population in mice is highly polyclonal. The repertoire is comprised of both highly expanded and low frequency clones, and with age new clones are introduced. Notably, expanded clones with previously selected specificities repopulated the small intestine after PC depletion and showed similar SHM frequencies, hence indicating the presence of a functional intestinal IgA memory compartment. A polyclonal and highly diverse IgA repertoire would parallel the broad range of intestinal antigens to which the intestinal mucosa is constantly exposed, although it also appears that the repertoire continuously adapts to the current composition of the microflora (Hapfelmeier et al., 2010). The high-throughput analysis of the intestinal IgA repertoire performed by Lindner et al. (2012) also suggested that clonal expansion is likely to occur predominantly in the periphery rather than locally in the LP, which has been a subject of debate in previous studies (Yuvaraj et al., 2009).

Taken together, these studies strongly indicate that the great majority of gut IgA antibodies develop from antigen-specific B cell responses, which evolve by acquisition of somatic mutations to confer effectiveness and high specificity.

### LONGEVITY OF INTESTINAL IgA PLASMA CELLS

Another matter of debate has been whether human intestinal IgA PCs can provide long-term humoral immunity. Evidence for a long-lived, commensal-specific IgA response was observed when germ-free mice were reversibly colonized by bacteria (Hapfelmeier et al., 2010). In absence of competition from newly generated cells, IgA PCs were shown to have a half-life of at least 16 weeks. The dynamic of such IgA response, however, reflected the contents of the intestinal lumen, suggesting that the number of long-lived PC niches is limited. As a consequence, in presence of competition such as that deriving from the continuously evolving microflora, “older” PCs – despite their long-lived potential – are constantly displaced by new ones, which are generated in response to the most recent stimuli. In agreement with these observations, we have demonstrated that the human small intestine harbors a population of non-proliferating PCs that are maintained by the local supportive microenvironment for long-term survival (Mesin et al., 2011). Moreover, an inflammatory microenvironment may enhance the niche capacity, resulting in more robust PC responses (Radbruch et al., 2006).

### MUCOSALLY INDUCED TOLERANCE

The homeostatic role of the intestinal immune system is to provide suppressed immune responses as to generate mucosally induced tolerance. Such tolerance can be directed toward orally administered antigens or toward gut bacteria. Thus, there are two layers of intestinal anti-inflammatory homeostatic mechanisms: immune exclusion of commensal bacteria by secretory antigen-specific IgA and immune suppression to avoid hypersensitivity to innocuous food antigens. These two mechanisms of mucosally induced tolerance appear to act independently in order to attenuate a broad range of immune responses (Weiner et al., 2011; Pabst and Mowat, 2012). The lack of such homeostatic tolerance results in intestinal immune pathology. Active proinflammatory immune responses directed toward the gut microbiota, inducing imbalance in IgA and IgG repertoires, are associate with the development of inflammatory bowel disease, such as Crohn’s disease and ulcerative colitis (Baklien and Brandtzaeg, 1976; Macpherson et al., 1996). In celiac disease (CD) there is an active proinflammatory immune response to cereal gluten antigens.

## CELIAC DISEASE

Celiac disease is a common intestinal disorder affecting 1% of the population in Europe and the US, although only a fraction of patients is readily diagnosed due to the highly variable clinical presentation of the disease (Green, 2005). CD can be considered a food intolerance to wheat gluten (consisting of the gliadin and glutenin subcomponents) and related proteins from rye and barley. In genetically predisposed individuals, gluten ingestion can cause an inflammatory reaction in the upper small intestine which gives tissue damage leading to villous atrophy (Sollid, 2002). The lesion and inflammatory changes disappear after weeks or months when patients for treatment purpose commence a gluten-free diet (GFD). The inflammatory reaction appears to be driven by activation of Th1-like CD4^+^ T cells (see **Box 1**) that recognize gluten peptides post-translationally modified by the enzyme transglutaminase 2 (TG2; Molberg et al., 1998; van de Wal et al., 1998). What initiates this “aggressive” T cell response and lack of oral tolerance to gluten is not known. It has been suggested that some part of gluten may have innate properties or that infections may play a role (reviewed in Jabri and Sollid, 2009). In steady-state conditions, the maintenance of intestinal homeostasis is initiated by intestinal DCs that are affected by enterocyte-derived factors, such as retinoic acid and TGF-β, conferring tolerogenic properties on the DCs. Tolerogenic DCs educate the intestinal immune system to respond in a non-inflammatory manner to orally administered proteins by the induction of regulatory T (Treg) cells. An alteration of the intestinal environment, as observed in CD, characterized by a high level of inflammatory cytokines such as IL-15 and IFNα, may affect the acquisition of the tolerogenic phenotype of intestinal DCs. This will prevent the induction of Treg cells, further promoting the differentiation of proinflammatory T cells (Jabri and Sollid, 2009). Interestingly, beside the strong gluten-specific T cell response, CD presents autoimmune features, most notably the production of autoantibodies. These antibodies are primarily directed against TG2 (Dieterich et al., 1997), but antibodies specific for other autoantigens like actin, collagen and others have also been described (Alaedini and Green, 2008). Whereas the T cell response to gluten has been thoroughly characterized and is relatively well understood, significantly less is known about the B cell responses in CD.

**Box 1. Immunopathogenesis of CD.**

CD is a multifactorial disease with a complex interplay between genetic and environmental factors eventually leading to chronic inflammation (see Abadie et al., 2011; Meresse et al., 2012 for review and references therein). Of these factors, the most significant genetic component is HLA; 90% of celiac patients carry a variant of DQ2 termed DQ2.5, whereas most of the remaining patients carry DQ8. The HLA association has been extensively investigated, and the study of lesion-derived T cell lines and T cell clones has allowed a detailed description of gluten T cell epitopes. Several epitopes exist and some epitopes are more frequently recognized than others (reviewed in Sollid et al., 2012). An important feature of both the DQ2.5 and DQ8 molecules is their preference for binding of negatively charged amino acid residues (i.e., glutamate or aspartate) in certain binding pockets (P4, P6, and P7 for DQ2.5; P1 and P9 for DQ8). Gluten proteins have very few negatively charged residues, however they carry a high amount of glutamine and proline residues. Interestingly, glutamine can be deamidated to glutamate by the enzyme TG2, and TG2-modified gluten peptides show strong immunogenicity. This suggests that, under particular circumstances, deamidation happens *in vivo*, leading to the formation of post-translationally modified gluten peptides that are suitable for presentation by DQ2.5 or DQ8 molecules. The gluten T cell epitopes are furthermore hallmarked by the presence of multiple proline residues, and this is particularly so for epitopes presented by DQ2.5. Proline, in addition to influencing MHC binding, exerts a force in the selection of T cell epitopes at two additional levels. First, peptides rich in proline are resistant to proteolysis, and proline-rich gluten peptides survive gastrointestinal digestion allowing them to reach the LP where they can by loaded on HLA-DQ molecules expressed by antigen-presenting cells. Second, proline guides the specificity of TG2 so that glutamine residues in the motif glutamine-X-proline are targeted. Notably, the peptides harboring T cell epitopes in a complex gluten digest are the preferred TG2 substrates. How these forces work can be visualized by looking at the α2-gliadin, a representative α-gliadin. Upon treatment with gastric and pancreatic endopeptidases a relatively large fragment, the 33-mer LQLQPFPQPQLPYPQPQLPYPQPQLPYPQPQPF (residues 57 to 89), survives digestion. Due to its length this peptide is resistant to digestion by small intestinal brush-border membrane ectopeptidases as well. Three glutamine residues within the 33-mer are deamidated by TG2. This deamidated 33-mer harbors six overlapping T cell epitopes and is an extremely potent antigen. Upon recognition of deamidated gluten peptides, the CD4^+^ T cells become activated and start producing cytokines including interferon-γ and interleukin-21. The priming of the gluten-specific CD4^+^ T cells likely takes place in GALT or mesenteric lymph nodes and the primed cells seed via the blood into the LP as effector cells. These T cells may be important for forming PC survival niches in the LP.

TG2 has several biological functions which include transamidation (cross-linking) and deamidation, and is involved in many physiological processes (Lorand and Graham, 2003). TG2 is present in large amounts in the gut LP, in particular in a sub-epithelial layer. It is hardly coincidental that TG2 is also the target of the autoantibodies in CD. Studies based on *in situ* detection with immunofluorescence (Korponay-Szabo et al., 2004) and phage display antibody libraries (Marzari et al., 2001) suggested that anti-TG2 antibodies are produced locally in the small intestine, and recently we were able to visualize the intestinal PCs producing such antibodies (Di Niro et al., 2012). In the following, we will describe the current knowledge and the future directions in the study of the intestinal B cell response in CD.

## THE INTESTINAL B CELL RESPONSE IN CD

The celiac lesion is characterized by considerable expansion of the PC population (Douglas et al., 1970; Soltoft, 1970) and enhanced local immunoglobulin secretion (Lancaster-Smith et al., 1974; Wood et al., 1987). In addition, there are IgA deposits at the epithelial basement membrane of the small intestine (Shiner and Ballard, 1972; Korponay-Szabo et al., 2004) which can be observed without overt histological changes (Salmi et al., 2006a). The plasmacytosis (increased median PCs per mucosal tissue unit of 2.1, 3.8, and 2.9-fold for IgA, IgM, and IgG respectively; Baklien et al., 1977; Scott et al., 1980) may relate to bolstering of a PC survival niche. Local plasmacytosis in CD appears to be homeostatic with an unaltered immunoglobulin isotype distribution and marked preponderance of IgA PCs (Brandtzaeg, 2006). Notably, the duodenal IgA PC population in active CD maintains mucosal phenotype by J-chain expression and consists of a higher proportion of the IgA2 subclass than in the normal duodenal mucosa (Kett et al., 1990). Upon dietary gluten restriction, intestinal PC numbers are reduced (Holmes et al., 1973).

### ANTI-GLIADIN AND ANTI-TG2 ANTIBODIES

Early experiments performed by ELISA, ELISpot, and immunofluorescence indicated local intestinal secretion of anti-gliadin antibodies (Stern and Dietrich, 1982; Ciclitira et al., 1986; Labrooy et al., 1986; Lycke et al., 1989). These studies suggested that gliadin-specific PCs account for 1–2, 10 and 5–10% of total IgA, IgM, and IgG PCs, respectively, in the small intestine of CD patients. Anti-gliadin IgA and IgG antibodies are detected in sera of untreated CD patients and can be harnessed as a diagnostic tool. These antibodies disappear after commencement of a GFD (Savilahti et al., 1983; Kilander et al., 1987), and they rise again when gluten is reintroduced into the diet (Koninckx et al., 1984). Thus, their level seems to mirror the immune reaction triggered by gluten in the intestine and, further, their decrease is related to a clinical improvement of the intestinal mucosa (Mayer et al., 1989; Valletta et al., 1990). IgA gliadin-specific B cells have been detected in peripheral blood of CD patients (Hansson et al., 1997; Sblattero et al., 2000); these possibly are circulating IgA plasmablasts homing to the LP.

Celiac disease patients also develop autoreactive antibodies originally identified as targeting connective tissue constituents, in particular the endomysium (Chorzelski et al., 1983). The enzyme TG2 was identified as the major endomysial autoantigen (Dieterich et al., 1997). In the diagnostic workup of CD, assessment of serum of anti-TG2 autoantibodies has become an important tool for the diagnosis particularly in children where new recommendations allow the diagnosis to be made without histological examination of small intestinal biopsies (Husby et al., 2012). Similarly to anti-gliadin antibodies, the production of anti-TG2 antibodies is dependent on dietary gluten exposure (Dieterich et al., 1998; Sulkanen et al., 1998). Anti-TG2 antibody titers have been shown to correlate with abnormal small intestine histopathology (Tursi et al., 2003). While serum anti-gliadin antibodies have a significant IgA2 component, only a minor portion of serum IgA antibodies reactive to the endomysium were found to belong to this subclass (Osman et al., 1996).

**FIGURE 1:**
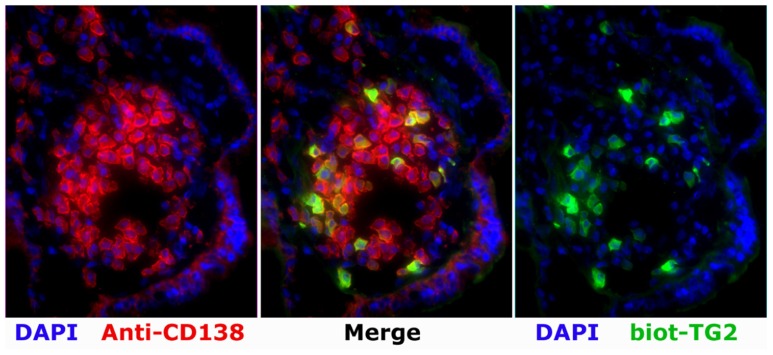
**A patch with high frequency of TG2-specific PCs as revealed by immunofluorescence analysis on a cryosection of the duodenal mucosa of a patient with active CD.** Staining performed with biotinylated TG2 (biot-TG2, followed by fluorescent streptavidin, *green*), a plasma cell marker (anti-CD138, *red*), and a nuclei stain (DAPI, *blue*).

Recently, we demonstrated that TG2-specific PCs can be visualized by immunofluorescence of tissue sections (**Figure 1**) and by flow cytometry of single-cell suspensions from duodenal biopsy specimens (Di Niro et al., 2012). To note, TG2-specific PCs comprise 4–24% of the total IgA PC population in the celiac lesion. This massive accumulation of TG2-specific PCs is further supported by the notion that IgA intestinal antibody deposits target the same antigen in the extracellular matrix and the endothelium of the small blood vessels (Korponay-Szabo et al., 2004), thus reflecting an abundant local antibody production. Notably, TG2-targeted IgA intestinal deposits are present at all stages of CD, including early developing CD (prior to villous atrophy; Kaukinen et al., 2005; Paparo et al., 2005; Tosco et al., 2008) as well as the advanced lesion stage in rare seronegative patients (Salmi et al., 2006b).

### CHARACTERISTICS OF THE ANTI-TG2 ANTIBODY REPERTOIRE

In our recent, thorough dissection of the antibody (Ab) repertoire of the intestinal autoimmune response to TG2, we found that despite extensive class switch to IgA, antibody genes had a very limited amount of somatic mutations (Di Niro et al., 2012). From the analysis of 60 sequences of heavy chain variable region genes cloned from single intestinal PCs of CD patients, we observed on average less than half the number of mutations found in the rest of the intestinal PC compartment. Interestingly, anti-TG2 antibodies with heavy chain encoded by the VH5 gene, which accounted for 44% of the response, were significantly less mutated than those encoded by other genes, including several that were entirely germ-line encoded. A trend of low mutation in VH5 genes in the gut was observed before (Boursier et al., 1999), but the reason for this and for the preferential recruitment of cells expressing antibodies encoded by VH5 in the autoimmune repertoire is not known. It may be related to structural properties of the VH5 region and/or to the fewer mutational hotspots in the VH5–51 gene. Worth noting is that a number of VH5 antibodies with different specificity did not bind TG2 to any extent, thus showing that the anti-TG2 reactivity is CDR-encoded and not depending on unspecific binding of the VH5 framework regions (Di Niro et al., 2012).

This significantly differs from all other intestinal human antibody repertoires described to date, as presented and discussed above, in which antibody genes with such a limited number of mutations were seldom observed. This represents a unique feature of the anti-TG2 response and may therefore provide clues into its generation.

### GENERATION OF TG2-SPECIFIC B CELLS

**FIGURE 2:**
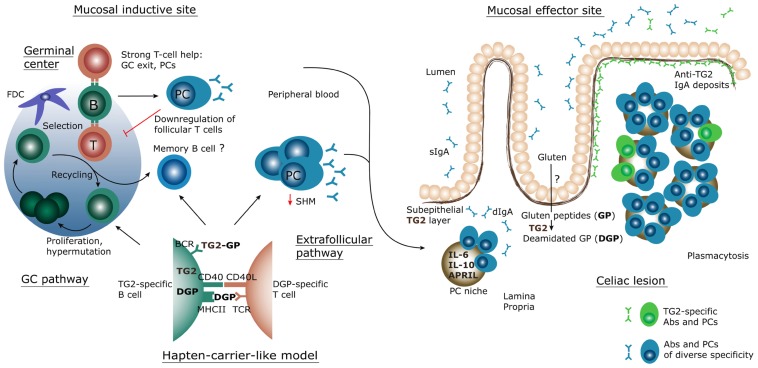
**Schematic representation of the inductive and effector sites for the mucosal B-cell response in CD.** Through yet unknown mechanisms, gluten peptides (GP) cross the epithelium and become deamidated gluten peptides (DGP) by the action of transglutaminase 2 (TG2). These peptides are then transported, possibly by dendritic cells, to local lymphoid tissue structures where a strong, proinflammatory T cell response takes place. Some native peptides may reach the lymphoid tissue and may become targeted by TG2 there. In lymphoid tissues TG2-specific B cells likely interact with gluten-specific T cells, according to the hapten-carrier-like model described in the text which builds on the ability of TG2 to form TG2-GP/DGP complexes. The B cell response generated follows either a canonical germinal center (GC) pathway or an extrafollicular pathway, resulting in massive plasmablast generation. The plasmablasts migrate via the blood to the lamina propria, where they mature as plasma cells (PC), secrete antibodies (Abs) and survive for extended time in what is called a PC survival niche. In active CD, this results in plasmacytosis and sustained secretion of IgA dimers (dIgA) – as well as IgM pentamers – which are transported across the epithelium to the lumen as secretory IgA (sIgA). TG2-specific antibodies produced at this site are also deposited on the subepithelial layer of TG2. BCR, B cell receptor; FDC, follicular dendritic cell; TCR, T cell receptor.

A schematization of the current knowledge and hypotheses for the generation of the intestinal TG2-specific B cell response is shown in **Figure 2**. The scheme assumes a T cell-dependent mechanism for generation of the response. Does instead the observed phenotype, with scarce SHM, indicate a T cell-independent one? Indeed, despite numerous efforts, to date there is no convincing evidence for the existence of TG2-specific T cells. However, the clinical observation of the strict HLA-dependent appearance of TG2-specific antibodies speaks against this model. In a population based cohort study, Bjorck et al. (2010) compared the presence of TG2-specific antibodies in children with or without the DQ2 or DQ8 HLA-risk alleles, and found that 73 of 1620 (4.5%) individuals with HLA-risk alleles were positive for anti-TG2 antibodies, versus none of 1815 subjects without HLA-risk alleles. This strongly suggests T cell involvement. How to reconcile these data with the inability to identify TG2-specific T cells? Once again, clinical observations provide clues: anti-TG2 antibodies rapidly disappear when gluten is removed from the diet (Dieterich et al., 1998; Sulkanen et al., 1998). The regulation of the B cell response by gluten intake is the foundation of the “hapten-carrier-like model” proposed several years ago (Sollid et al., 1997). In this model, intestinal gluten-specific T cells provide help to TG2-specific B cells. This is possible because of the enzymatic activity of TG2. The enzyme can form covalently linked complexes between itself and gluten peptides (Fleckenstein et al., 2004), which then can act as “hapten-carrier-like” complexes. Upon withdrawal of gluten from the diet, the T cell help will cease and anti-TG2 antibodies will disappear. Although this model has not been formally demonstrated *in vivo*, we recently provided *in vitro* evidence that TG2-specific B cells indeed can present gluten peptides to gluten-reactive T cells when offered TG2–gluten peptide complexes (Di Niro et al., 2012).

Based on strong clinical evidence, T cells thus appear to be involved in the generation of the anti-TG2 B cell response. In what is regarded as the “canonical” response, T and B cells cooperate in the GC, where B cells not only class-switch, but also accumulate mutations so that clones with increased affinity are selected (affinity maturation) in a process that ultimately generates memory B cells and long-lived PCs. In particular, B cell memory is “designed” to respond more rapidly to a secondary antigen challenge, and repeated stimulation leads to the generation of a switched response composed of high affinity clones which have acquired many mutations. In humans, this is clear in the cases of HIV, influenza, and rotavirus infections (Wrammert et al., 2008; Scheid et al., 2009; Di Niro et al., 2010).

A possible scenario to explain the phenotype of the anti-TG2 response is that it is generated at extrafollicular sites, thus bypassing GC formation (MacLennan et al., 2003) but nonetheless maintaining the requirement for T cell involvement. In mice, this has been observed in more than one context, such as *Salmonella* infection (Cunningham et al., 2007) and, interestingly, the autoimmune RF response (William et al., 2002). Contrary to what commonly thought, AID can be expressed at EF sites (Marshall et al., 2011), and in the autoimmune context SHM has clearly been shown to vigorously take place at this site (William et al., 2002). The intestinal environment may play a role in this process. AID-dependent class switching to IgA at the LP level has been shown in mice (Fagarasan et al., 2001) and studies in humans suggested that proliferation, class-switching, and SHM could take place in the intestinal LP (He et al., 2007; Yuvaraj et al., 2009). As discussed above, a study of mice based on analysis of IgA sequences obtained by high-throughput sequencing suggested instead that extra-mucosal expansion is followed by massive seeding in the LP, of which clonal relatedness is a consequence (Lindner et al., 2012). Although still being a matter of major debate, it cannot be excluded that the TG2-specific response expands and evolves in the LP, and that B cells do not enter GCs at all. If they do, the scarce number of mutations suggests that they undergo limited rounds of selection, possibly a single one, in the GC. The scope of the GC is to ultimately produce memory cells and long-lived PCs. Does this happen in the case of the anti-TG2 response? For both cell populations evidence is limited and further studies are required. Upon gluten removal the TG2-specific IgA serum titer decreases within months (Maki et al., 1998), suggesting that the long-lived PC compartment is limited. Although finally differentiated to a PC phenotype, IgA PCs in the small intestine are only relatively long-lived, with a lifespan of some months (Mesin et al., 2011); the microenvironment is likely to play a major role. Similarly, we have only partial evidence for the existence of a TG2-specific, IgA memory compartment in CD, which appears to be more substantial in GFD-treated rather than active CD patients. It has to be noted that small amounts of memory B cells seem to be produced also in the absence of GCs (Toyama et al., 2002; Foote and Kearney, 2009).

### POSSIBLE REGULATION MECHANISMS OF THE ANTI-TG2 IMMUNE RESPONSE

Whether cells are or not generated in GCs, undoubtedly specific factors limit GC activity. To note, the diagnosis of CD (and thus specimen sampling) often occurs many months or years after the appearance of the symptoms. Moreover, the anti-TG2 response precedes symptoms and intestinal damage. All the patients from whom we obtained intestinal specimens were adults (Di Niro et al., 2012); the anti-TG2 response had likely been present for several months or years. In this time frame, according to the canonical model of immune response, GCs should be formed, memory generated, upon secondary stimulation memory cells preferentially and rapidly reactivated, resulting in extensive affinity maturation and accumulation of mutations. What inhibits this? Our current knowledge only allows speculation. Several different, and not mutually exclusive, scenarios can be envisaged.

(i) Feedback mechanisms. These could happen both at the antibody and the antigen level, as well as at the cellular level. The extent of the anti-TG2 response (10% of intestinal PCs, on average) and the consequent massive antibody production may provide a negative feedback that inhibits GC activity. This could be seen as a self-regulating mechanism of the immune system. With regard to antigen availability, chronic stimulation (i.e., continuous gluten ingestion) may be different than repeated antigen challenge (i.e., seasonal flu infections). Another feedback mechanism may result from antigen-specific, isotype-switched PCs that before homing to the LP act as potent antigen presenting cells. PC-controlled antigen presentation was shown to suppress the development and function of antigen-specific follicular helper T cells (Pelletier et al., 2010). Such function of PCs would create bidirectional regulation of adaptive immunity by providing a negative cognate regulatory mechanism which may serve as a functional sensor of PC production that can control ongoing GC B cell responses.

(ii) Affinity. The binding strength of the anti-TG2 antibodies may be a factor. Even when a panel of mutated anti-TG2 hmAbs was reverted to their germline counterparts, strong binding to TG2 was observed (Di Niro et al., 2012). This is atypical as in the case of anti-flu antibodies removal of mutations gave a dramatic loss in affinity (Di Niro et al., 2012). It has been previously shown in mice that high affinity may favor EF T cell-dependent PC responses (Chan et al., 2009). Alternatively, it may be that inside GCs it is affinity that regulates the fate of B cells – i.e., whether to become a memory B cell or PC – and that high affinity favors the latter. In both scenarios, this would result in generation of a scarce memory compartment and, irrespective of the specific mechanism, to continuous activation of naïve B cells thus explaining scarce SHM. As an alternative explanation, it has been shown that the initial affinity of the B cell receptor (BCR) is inversely correlated with accumulation of mutations in GC T cell-dependent antibody responses *in vivo* (Shih et al., 2002). This effect was due to GC selection, as both high and low affinity B cells had the same frequency of mutations in non-coding sequences.

(iii) T cell control. T cells may represent an important regulator. It has been shown that a robust and efficient T cell response increases the magnitude of the PC response while preventing GC recycling and memory cell differentiation (Fujihashi et al., 1991; Bolduc et al., 2010; Heiser et al., 2011). Furthermore, it has been suggested that the decision to become a PC upon receiving T cell help is antigen dose-dependent (Victora and Nussenzweig, 2012). If the help to TG2-specific B cells indeed is provided by gliadin-specific T cells, the amount of antigen and strength of the T cell response would be compatible with a limited GC reaction.

(iv) Nature and location of the response. The nature (autoimmune) and the location (intestine) differ from any other B cell responses that have been characterized in humans, and hence models for comparison are not easily available. In mice, peritoneal reservoirs of B1 cells significantly contribute to intestinal responses (Kroese et al., 1995) – often via T cell-independent mechanisms, that do not efficiently form GCs (Toellner et al., 2002). Whether similar mechanisms take place in humans is not fully understood (Griffin and Rothstein, 2012). Could instead the unique features of the anti-TG2 response relate to the self nature of the antigenic target? Does the immune system sense the antigen as self and redirects the response toward an EF one? Germ-line encoded autoreactivity has been described, for instance in RA (Victor et al., 1991), but does not seem to be the general rule. In mice, there are notable examples of autoimmune responses developing at EF sites – the response to RF being such one situation (William et al., 2002). Important insights will come from the analysis of the repertoire of the gluten-specific B cell response. Would gluten-specific PCs show SHM at the same level as TG2-specific PCs, or would they have high degrees of SHM as generally seen in intestinal IgA PCs? Together with the investigation of the CD-specific B cell memory, this is one of the most interesting immunological aspects toward which research should focus.

(v) Structural features. As mentioned above, anti-TG2 antibodies have high affinity even when germ- line encoded. What confers such high affinity and, in particular, what favors VH5 selection over other V regions is not known. On the surface of a B cell, BCR cross-linking is one potent mechanism for activation. We recently showed that, *in vitro*, TG2 can mediate covalent cross-linking of IgD (and, to a minor extent, IgM, but not of IgA or IgG) antibodies, providing a hypothetical model where continuous activation of naïve B cells is favored over IgA-switched memory cells, thus explaining lack of accumulated mutations (Di Niro et al., 2012). Similarly, it is conceivable that the VH5 dominance is based on a similar mechanism.

To address these questions, we need to know more about how antibodies bind to TG2. The hmAbs that we have cloned from intestinal IgA PCs represent a unique tool to better understand their interaction with TG2. We are currently investigating the epitopes recognized by the autoantibodies, as well as their ability to bind TG2 in its different forms (i.e., free vs bound to fibronectin, open vs close conformation, GTP vs Ca^2^^+^-bound, etc). Ultimately, fundamental insights will derive from efforts directed toward the generation of crystal structures of TG2-hmAb complexes.

### TG2-SPECIFIC B CELLS AS ANTIGEN PRESENTING CELLS AMPLIFYING THE ANTI-GLUTEN T CELL RESPONSE

B cells can program CD4^+^ T cell responses (reviewed in Barr et al., 2012). This is so, much because B cells and T cells interact in an antigen-specific manner. By BCR-mediated uptake and concentration of antigen, B cells serve as potent antigen-presenting cells for T cells (Lanzavecchia, 1985). This mechanism is essential for B cells to receive cognate T cell help, but notably it has also direct consequences for the T cells. B cell-mediated antigen presentation leads to proliferation and clonal expansion of antigen-specific T cells (Crawford et al., 2006). If TG2-specific B cells are able to load deamidated gluten peptides for presentation to gluten-specific T cells *in vivo* (Sollid et al., 1997), this would likely result in expansion of gluten-specific T cell clones. These gluten-specific T cells would then be able to interact both with TG2-specific B cells as well as with B cells specific for deamidated gluten peptides. Collectively, these events will support the antibody responses to TG2 and deamidated gluten peptides and importantly lead to an amplification of the anti-gluten T cell response. In this scenario, B cells would be at the center stage of the immunopathogenesis of CD, and could therefore be a potential target for therapy even if CD is considered primarily a T cell-mediated disease.

### PATHOGENIC ROLE AND INTERACTION OF AUTOANTIBODIES WITH INTESTINAL STRUCTURES

Since the discovery of autoantibodies in CD, and subsequently the identification of TG2 as the main target, there has been speculation about whether the antibodies themselves are pathogenic. Most research has focused on the effects of antibodies on enzymatic activity, with discordant results (Esposito et al., 2002; Dieterich et al., 2003; Di Niro et al., 2012). A reason for this could be the variety of assays and experimental conditions used to assess TG2 activity. Although in some cases weak inhibition has been reported, as of now there is no compelling evidence that the effects of autoantibodies on TG2 play a major role, either in the pathogenesis or in relation to the clinical features of CD. The great majority of the hmAbs from our newly generated panel neither inhibited nor enhanced TG2 activity, consistent with a central role for TG2 in the enzymatic deamidation of gluten peptides.

Among other proposed effects, anti-TG2 antibodies could contribute to the formation of the lesion by inhibiting angiogenesis (Myrsky et al., 2008) as well as by interfering with the differentiation of epithelial cells (Halttunen and Maki, 1999). Effector function of antibodies could contribute to the tissue damage seen in CD. Complement-dependent inflammation has been observed in the CD lesion (Halstensen et al., 1992). Unlike IgA, IgM does fix the complement, and TG2-specific IgM are indeed produced in patients, especially in those with IgA deficiency (Borrelli et al., 2010; Di Niro et al., 2012).

Celiac disease patients with active disease have increased transport of gliadin peptides across the epithelium (Schumann et al., 2008), and IgA antibodies have also been suggested to have a role in such transport (Rauhavirta et al., 2011). In the intestinal mucosa TG2 binds fibronectin, forming a sub-epithelial layer, and in patients IgA/IgM deposits are observed at this location. Moreover, IgA is found on the brush border. Matysiak-Budnik et al. (2008) have shown that gliadin peptides can be retro-transcytosed as IgA–gliadin complexes via the transferrin receptor, which is abnormally expressed at the apical surface of enterocytes in active CD. TG2 localized at the apical side of the epithelium may as well play a role in this mechanism (Lebreton et al., 2012). In the LP, intact immunostimulatory gliadin peptides might act by triggering a local immune response and promoting inflammation. We hypothesize that dimeric anti-TG2 IgA play a role in this mechanism. Anti-TG2 antibodies could detach TG2 from fibronectin and the complexes could be transported across the epithelium, where sampling of gliadin peptides by TG2 itself or by anti-gliadin antibodies could take place. We have preliminary evidence that a fraction of anti-TG2 antibodies can in fact compete for the fibronectin binding site.

In conclusion, as of now the evidence for a role of anti-TG2 antibodies is scarce, and it derives from *in vitro *or cell culture systems; when anti-TG2 antibodies were expressed *in vivo* in mice (Di Niro et al., 2008), no obvious effect was seen. This highlights the need for the generation of an animal model of CD.

### ANIMAL MODELS OF CD

Several animal models have been developed that try to recapitulate CD, however none of them entirely succeeded reproducing the complex mechanisms causing this disease (reviewed in Marietta and Murray, 2012). Expression of anti-TG2 autoantibodies *in vivo* by means of adeno-associated virus-based gene transfer led to lifetime production of such antibodies, analogous to what observed in patients, but no clinical features were associated (Di Niro et al., 2008). Some signs of disease were obtained when pre-sensitized CD4^+^ T cells were transferred in Rag-deficient mice, inducing weight loss and duodenitis (Freitag et al., 2009); however also this system was not able to recapitulate the majority of the immune features observed in CD. Interestingly, Jabri’s group has described a humanized HLA-DQ8-transgenic mouse model (DePaolo et al., 2011), characterized by over-expression of IL-15 in the LP. When gliadin-fed, these animals develop IFN-γ-producing anti-gliadin T cells, anti-gliadin and anti-TG2 antibodies, and intraepithelial lymphocytosis. Despite lacking the hallmark of villous atrophy, this model does resemble early stages of CD. Mice are not the only species where CD models are investigated, as a screening of macaques also led to identification of animals with signs and symptoms of CD (Bethune et al., 2008); the usefulness of such model remains to be evaluated. In general, efforts are being made toward the generation of an animal model of CD, which will greatly facilitate research.

### THE MICROFLORA AND ITS IMPACT ON INTESTINAL IMMUNITY

Genetic factors may account only for about half of the risk to develop CD thus leaving an important role for the environment in the pathogenesis. Gluten exposure obviously is critical, but environmental factors outside of gluten may be implicated as well. Such factors could be pathogenic infectious agents or commensal bacteria. Recently, it has become clear that the gut microflora profoundly influence intestinal immunity (Kau et al., 2011). There is a complex interplay between intestinal immunity and the populations of commensal bacteria, and these two components regulate each other. Not only does the microflora regulate several aspects of the innate and adaptive immunity, as well as of several metabolic pathways, but it has also been shown that dysregulated immunity (for instance as a consequence of experimental manipulation of molecules such as PD-1 and AID; Wei et al., 2011; Kawamoto et al., 2012) results in skewed gut microbial communities, and this in turn may have detrimental effects. In future, it will be of major importance to understand how the microbial communities contribute to the intestinal immune response in a context such as CD where both the T and the B cell intestinal populations seem altered as compared to healthy individuals.

## CONCLUSION

In the last two decades we have learnt a lot about CD and its interplay with intestinal immunity. Among the most remarkable discoveries, TG2 has been identified as the main autoantigen of CD, and its role in creating potent T cell epitopes has been unraveled. We have made huge steps forward in understanding the role of HLA genes, and many non-HLA susceptibility genes have been identified. Some limited progress has also been made in understanding the role of innate immunity factors in CD. Recently, the knowledge of the intestinal B cell response in CD has significantly improved. We have learnt that anti-TG2 antibodies form deposits in the small intestine, a number of highly sensitive serological tests based on serum antibodies have been developed, monoclonal antibodies have been isolated by phage display and single cell cloning, and the cells producing these antibodies have been visualized, characterized and enumerated. However, many important features of CD, in particular related to the intestinal environment in which the disease takes place, remain to be tackled. These include characterization of the anti-gliadin B-cell response, the IgG anti-TG2 repertoire, identification of the memory compartment, and others. A better understanding of human intestinal immunobiology is needed to address these questions.

## Conflict of Interest Statement

The authors declare that the research was conducted in the absence of any commercial or financial relationships that could be construed as a potential conflict of interest.

## References

[B1] Abadie V. Sollid L. M. Barreiro L. B. Jabri B. (2011). Integration of genetic and immunological insights into a model of celiac disease pathogenesis. *Annu. Rev. Immunol.* 29 493–525 21219178 10.1146/annurev-immunol-040210-092915

[B2] Alaedini A. Green P. H. (2008). Autoantibodies in celiac disease. *Autoimmunity* 41 19–26 18176861 10.1080/08916930701619219

[B3] Baklien K. Brandtzaeg P. (1976). Immunohistochemical characterization of local immunoglobulin formation in Crohn’s disease of the ileum. *Scand. J. Gastroenterol.* 11 447–457 785586

[B4] Baklien K. Brandtzaeg P. Fausa O. (1977). Immunoglobulins in jejunal mucosa and serum from patients with adult coeliac disease. *Scand. J. Gastroenterol.* 12 149–159 322252

[B5] Barone F. Patel P. Sanderson J. D. Spencer J. (2009). Gut-associated lymphoid tissue contains the molecular machinery to support T-cell-dependent and T-cell-independent class switch recombination. *Mucosal Immunol.* 2 495–503 19741596 10.1038/mi.2009.106

[B6] Barone F. Vossenkamper A. Boursier L. Su W. Watson A. John S. (2011). IgA-producing plasma cells originate from germinal centers that are induced by B-cell receptor engagement in humans. *Gastroenterology* 140 947–956 21147106 10.1053/j.gastro.2010.12.005 PMC7115992

[B7] Barr T. A. Gray M. Gray D. (2012). B cells: programmers of CD4 T cell responses. *Infect. Disord. Drug Targets* 12 222–231 22394172 10.2174/187152612800564446

[B8] Bemark M. Boysen P. Lycke N. Y. (2012). Induction of gut IgA production through T cell-dependent and T cell-independent pathways. *Ann. N. Y. Acad. Sci.* 1247 97–116 22260403 10.1111/j.1749-6632.2011.06378.x

[B9] Benckert J. Schmolka N. Kreschel C. Zoller M. J. Sturm A. Wiedenmann B. (2011). The majority of intestinal IgA+ and IgG+ plasmablasts in the human gut are antigen-specific. *J. Clin. Invest.* 121 1946–1955 21490392 10.1172/JCI44447 PMC3083800

[B10] Benson M. J. Dillon S. R. Castigli E. Geha R. S. Xu S. Lam K. P. (2008). Cutting edge: the dependence of plasma cells and independence of memory B cells on BAFF and APRIL. *J. Immunol.* 180 3655–3659 18322170 10.4049/jimmunol.180.6.3655

[B11] Bergqvist P. Gardby E. Stensson A. Bemark M. Lycke N. Y. (2006). Gut IgA class switch recombination in the absence of CD40 does not occur in the lamina propria and is independent of germinal centers. *J. Immunol.* 177 7772–7783 17114448 10.4049/jimmunol.177.11.7772

[B12] Bergqvist P. Stensson A. Lycke N. Y. Bemark M. (2010). T cell-independent IgA class switch recombination is restricted to the GALT and occurs prior to manifest germinal center formation. *J. Immunol.* 184 3545–3553 20207993 10.4049/jimmunol.0901895

[B13] Bethune M. T. Borda J. T. Ribka E. Liu M. X. Phillippi-Falkenstein K. Jandacek R. J. (2008). A non-human primate model for gluten sensitivity. *PLoS ONE* 3 e1614 10.1371/journal.pone.0001614 PMC2229647 18286171

[B14] Bjorck S. Brundin C. Lorinc E. Lynch K. F. Agardh D. (2010). Screening detects a high proportion of celiac disease in young HLA-genotyped children. *J. Pediatr. Gastroenterol. Nutr.* 50 49–53 19915493 10.1097/MPG.0b013e3181b477a6

[B15] Bolduc A. Long E. Stapler D. Cascalho M. Tsubata T. Koni P. A. (2010). Constitutive CD40L expression on B cells prematurely terminates germinal center response and leads to augmented plasma cell production in T cell areas. *J. Immunol.* 185 220–230 20505142 10.4049/jimmunol.0901689 PMC3708602

[B16] Borrelli M. Maglio M. Agnese M. Paparo F. Gentile S. Colicchio B. (2010). High density of intraepithelial gammadelta lymphocytes and deposits of immunoglobulin (Ig)M anti-tissue transglutaminase antibodies in the jejunum of coeliac patients with IgA deficiency. *Clin. Exp. Immunol.* 160 199–206 20030673 10.1111/j.1365-2249.2009.04077.x PMC2857942

[B17] Boursier L. Dunn-Walters D. K. Spencer J. (1999). Characteristics of IgVH genes used by human intestinal plasma cells from childhood. *Immunology* 97 558–564 10457207 10.1046/j.1365-2567.1999.00843.x PMC2326878

[B18] Boursier L. Gordon J. N. Thiagamoorthy S. Edgeworth J. D. Spencer J. (2005). Human intestinal IgA response is generated in the organized gut-associated lymphoid tissue but not in the lamina propria. *Gastroenterology* 128 1879–1889 15940623 10.1053/j.gastro.2005.03.047

[B19] Brandtzaeg P. (2006). The changing immunological paradigm in coeliac disease. *Immunol. Lett.* 105 127–139 16647763 10.1016/j.imlet.2006.03.004

[B20] Brandtzaeg P. Farstad I. N. Johansen F. E. Morton H. C. Norderhaug I. N. Yamanaka T. (1999). The B-cell system of human mucosae and exocrine glands. *Immunol. Rev.* 171 45–87 10582165 10.1111/j.1600-065X.1999.tb01342.x PMC7159139

[B21] Brandtzaeg P. Johansen F. E. (2005). Mucosal B cells: phenotypic characteristics, transcriptional regulation, and homing properties. *Immunol. Rev.* 206 32–63 16048541 10.1111/j.0105-2896.2005.00283.x

[B22] Brandtzaeg P. Pabst R. (2004). Let’s go mucosal: communication on slippery ground. *Trends Immunol.* 25 570–577 15489184 10.1016/j.it.2004.09.005

[B23] Brandtzaeg P. Prydz H. (1984). Direct evidence for an integrated function of J chain and secretory component in epithelial transport of immunoglobulins. *Nature* 311 71–73 6433206 10.1038/311071a0

[B24] Casola S. Otipoby K. L. Alimzhanov M. Humme S. Uyttersprot N. Kutok J. L. (2004). B cell receptor signal strength determines B cell fate. *Nat. Immunol.* 5 317–327 14758357 10.1038/ni1036

[B25] Cerutti A. (2008). The regulation of IgA class switching. *Nat. Rev. Immunol.* 8 421–434 18483500 10.1038/nri2322 PMC3062538

[B26] Cerutti A. Chen K. Chorny A. (2011). Immunoglobulin responses at the mucosal interface. *Annu. Rev. Immunol.* 29 273–293 21219173 10.1146/annurev-immunol-031210-101317 PMC3064559

[B27] Cerutti A. Rescigno M. (2008). The biology of intestinal immunoglobulin A responses. *Immunity* 28 740–750 18549797 10.1016/j.immuni.2008.05.001 PMC3057455

[B28] Chan T. D. Gatto D. Wood K. Camidge T. Basten A. Brink R. (2009). Antigen affinity controls rapid T-dependent antibody production by driving the expansion rather than the differentiation or extrafollicular migration of early plasmablasts. *J. Immunol.* 183 3139–3149 19666691 10.4049/jimmunol.0901690

[B29] Chorzelski T. P. Sulej J. Tchorzewska H. Jablonska S. Beutner E. H. Kumar V. (1983). IgA class endomysium antibodies in dermatitis herpetiformis and coeliac disease. *Ann. N. Y. Acad. Sci.* 420 325–334 6586098 10.1111/j.1749-6632.1983.tb22220.x

[B30] Ciclitira P. J. Ellis H. J. Wood G. M. Howdle P. D. Losowsky M. S. (1986). Secretion of gliadin antibody by coeliac jejunal mucosal biopsies cultured *in vitro*. *Clin. Exp. Immunol.* 64 119–124 3731523 PMC1542153

[B31] Crabbe P. A. Nash D. R. Bazin H. Eyssen D. V. Heremans J. F. (1969). Antibodies of the IgA type in intestinal plasma cells of germfree mice after oral or parenteral immunization with ferritin. *J. Exp. Med.* 130 723–744 4186443 10.1084/jem.130.4.723 PMC2138719

[B32] Crawford A. MacLeod M. Schumacher T. Corlett L. Gray D. (2006). Primary T cell expansion and differentiation *in vivo* requires antigen presentation by B cells. *J. Immunol.* 176 3498–3506 16517718 10.4049/jimmunol.176.6.3498

[B33] Crouch E. E. Li Z. Takizawa M. Fichtner-Feigl S. Gourzi P. Montano C. (2007). Regulation of AID expression in the immune response. *J. Exp. Med.* 204 1145–1156 17452520 10.1084/jem.20061952 PMC2118564

[B34] Cunningham A. F. Gaspal F. Serre K. Mohr E. Henderson I. R. Scott-Tucker A. (2007). Salmonella induces a switched antibody response without germinal centers that impedes the extracellular spread of infection. *J. Immunol.* 178 6200–6207 17475847 10.4049/jimmunol.178.10.6200

[B35] DePaolo R. W. Abadie V. Tang F. Fehlner-Peach H. Hall J. A. Wang W. (2011). Co-adjuvant effects of retinoic acid and IL-15 induce inflammatory immunity to dietary antigens. *Nature* 471 220–224 21307853 10.1038/nature09849 PMC3076739

[B36] Descatoire M. Weill J. C. Reynaud C. A. Weller S. (2011). A human equivalent of mouse B-1 cells? *J.Exp. Med.* 208 2563–2564; author reply 2566–2569. 22184680 10.1084/jem.20112232 PMC3244035

[B37] Di Niro R. Mesin L. Raki M. Zheng N. Y. Lund-Johansen F. Lundin K. E. A. (2010). Rapid generation of rotavirus-specific human monoclonal antibodies from small intestinal mucosa. *J. Immunol.* 185 5377–5383 20935207 10.4049/jimmunol.1001587

[B38] Di Niro R. Mesin L. Zheng N. Y. Stamnaes J. Morrissey M. Lee J. H. (2012). High abundance of plasma cells secreting transglutaminase 2-specific IgA autoantibodies with limited somatic hypermutation in celiac disease intestinal lesions. *Nat. Med.* 18 441–445 22366952 10.1038/nm.2656 PMC4533878

[B39] Di Niro R. Sblattero D. Florian F. Stebel M. Zentilin L. Giacca M. (2008). Anti-idiotypic response in mice expressing human autoantibodies. *Mol. Immunol.* 45 1782–1791 17996305 10.1016/j.molimm.2007.09.025

[B40] Dieterich W. Ehnis T. Bauer M. Donner P. Volta U. Riecken E. O. (1997). Identification of tissue transglutaminase as the autoantigen of celiac disease. *Nat. Med.* 3 797–801 9212111 10.1038/nm0797-797

[B41] Dieterich W. Laag E. Schopper H. Volta U. Ferguson A. Gillett H. (1998). Autoantibodies to tissue transglutaminase as predictors of celiac disease. *Gastroenterology* 115 1317–1321 9834256 10.1016/s0016-5085(98)70007-1

[B42] Dieterich W. Trapp D. Esslinger B. Leidenberger M. Piper J. Hahn E. (2003). Autoantibodies of patients with coeliac disease are insufficient to block tissue transglutaminase activity. *Gut* 52 1562–1566 14570723 10.1136/gut.52.11.1562 PMC1773855

[B43] Douglas A. P. Crabbe P. A. Hobbs J. R. (1970). Immunochemical studies on the serum, intestinal secretions and intestinal mucosa in patients with adult celiac disease and other forms of the celiac syndrome. *Gastroenterology* 59 414–425 4195762

[B44] Dunn-Walters D. K. Boursier L. Spencer J. (1997). Hypermutation, diversity and dissemination of human intestinal lamina propria plasma cells. *Eur. J. Immunol.* 27 2959–2964 9394824 10.1002/eji.1830271131

[B45] Elgueta R. Benson M. J. De Vries V. C. Wasiuk A. Guo Y. Noelle R. J. (2009). Molecular mechanism and function of CD40/CD40L engagement in the immune system. *Immunol. Rev.* 229 152–172 19426221 10.1111/j.1600-065X.2009.00782.x PMC3826168

[B46] Esposito C. Paparo F. Caputo I. Rossi M. Maglio M. Sblattero D. (2002). Anti-tissue transglutaminase antibodies from coeliac patients inhibit transglutaminase activity both *in vitro* and *in situ*. *Gut* 51 177–181 12117875 10.1136/gut.51.2.177 PMC1773330

[B47] Fagarasan S. Honjo T. (2000). T-Independent immune response: new aspects of B cell biology. *Science* 290 89–92 11021805 10.1126/science.290.5489.89

[B48] Fagarasan S. Kawamoto S. Kanagawa O. Suzuki K. (2010). Adaptive immune regulation in the gut: T cell-dependent and T cell-independent IgA synthesis. *Annu. Rev. Immunol.* 28 243–273 20192805 10.1146/annurev-immunol-030409-101314

[B49] Fagarasan S. Kinoshita K. Muramatsu M. Ikuta K. Honjo T. (2001). *In situ* class switching and differentiation to IgA-producing cells in the gut lamina propria. *Nature* 413 639–643 11675788 10.1038/35098100

[B50] Farstad I. N. Carlsen H. Morton H. C. Brandtzaeg P. (2000). Immunoglobulin A cell distribution in the human small intestine: phenotypic and functional characteristics. *Immunology* 101 354–363 11106939 10.1046/j.1365-2567.2000.00118.x PMC2327091

[B51] Fayette J. Dubois B. Vandenabeele S. Bridon J. M. Vanbervliet B. Durand I. (1997). Human dendritic cells skew isotype switching of CD40-activated naive B cells towards IgA1 and IgA2. *J. Exp. Med.* 185 1909–1918 9166420 10.1084/jem.185.11.1909 PMC2196343

[B52] Ferrari S. Giliani S. Insalaco A. Al-Ghonaium A. Soresina A. R. Loubser M. (2001). Mutations of CD40 gene cause an autosomal recessive form of immunodeficiency with hyper IgM. *Proc. Natl. Acad. Sci. U.S.A.* 98 12614–12619 11675497 10.1073/pnas.221456898 PMC60102

[B53] Fleckenstein B. Qiao S. W. Larsen M. R. Jung G. Roepstorff P. Sollid L. M. (2004). Molecular characterization of covalent complexes between tissue transglutaminase and gliadin peptides. *J. Biol. Chem.* 279 17607–17616 14747475 10.1074/jbc.M310198200

[B54] Foote J. B. Kearney J. F. (2009). Generation of B cell memory to the bacterial polysaccharide alpha-1,3 dextran. *J. Immunol.* 183 6359–6368 19841173 10.4049/jimmunol.0902473 PMC3235742

[B55] Freitag T. L. Rietdijk S. Junker Y. Popov Y. Bhan A. K. Kelly C. P. (2009). Gliadin-primed CD4+CD45RBlowCD25- T cells drive gluten-dependent small intestinal damage after adoptive transfer into lymphopenic mice. *Gut* 58 1597–1605 19671544 10.1136/gut.2009.186361 PMC3733237

[B56] Fujihashi K. McGhee J. R. Lue C. Beagley K. W. Taga T. Hirano T. (1991). Human appendix B cells naturally express receptors for and respond to interleukin 6 with selective IgA1 and IgA2 synthesis. *J. Clin. Invest.* 88 248–252 2056119 10.1172/JCI115284 PMC296026

[B57] Gibbons D. L. Spencer J. (2011). Mouse and human intestinal immunity: same ballpark, different players; different rules, same score. *Mucosal Immunol.* 4 148–157 21228770 10.1038/mi.2010.85

[B58] Gonnella P. A. Chen Y. Inobe J. Komagata Y. Quartulli M. Weiner H. L. (1998). *In situ* immune response in gut-associated lymphoid tissue (GALT) following oral antigen in TCR-transgenic mice. *J. Immunol.* 160 4708–4718 9590216

[B59] Green P. H. (2005). The many faces of celiac disease: clinical presentation of celiac disease in the adult population. *Gastroenterology* 128 S74–S78 15825130 10.1053/j.gastro.2005.02.016

[B60] Griffin D. O. Rothstein T. L. (2012). Human B1 cell frequency: isolation and analysis of human B1 cells. *Front. Immunol.* 3:122 10.3389/fimmu.2012.00122 PMC3360193 22654880

[B61] Guy-Grand D. Griscelli C. Vassalli P. (1975). Peyer’s patches, gut IgA plasma cells and thymic function: study in nude mice bearing thymic grafts. *J. Immunol.* 115 361–364 807633

[B62] Halstensen T. S. Hvatum M. Scott H. Fausa O. Brandtzaeg P. (1992). Association of subepithelial deposition of activated complement and immunoglobulin G and M response to gluten in celiac disease. *Gastroenterology* 102 751–759 1537512 10.1016/0016-5085(92)90155-r

[B63] Halttunen T. Maki M. (1999). Serum immunoglobulin A from patients with celiac disease inhibits human T84 intestinal crypt epithelial cell differentiation. *Gastroenterology* 116 566–572 10029615 10.1016/s0016-5085(99)70178-2

[B64] Hansson T. Dannaeus A. Kraaz W. Sjoberg O. Klareskog L. (1997). Production of antibodies to gliadin by peripheral blood lymphocytes in children with celiac disease: the use of an enzyme-linked immunospot technique for screening and follow-up. *Pediatr. Res.* 41 554–559 9098859 10.1203/00006450-199704000-00016

[B65] Hapfelmeier S. Lawson M. A. Slack E. Kirundi J. K. Stoel M. Heikenwalder M. (2010). Reversible microbial colonization of germ-free mice reveals the dynamics of IgA immune responses. *Science* 328 1705–1709 20576892 10.1126/science.1188454 PMC3923373

[B66] Harwood N. E. Batista F. D. (2010). Early events in B cell activation. *Annu. Rev. Immunol.* 28 185–210 20192804 10.1146/annurev-immunol-030409-101216

[B67] He B. Xu W. Santini P. A. Polydorides A. D. Chiu A. Estrella J. (2007). Intestinal bacteria trigger T cell-independent immunoglobulin A(2) class switching by inducing epithelial-cell secretion of the cytokine APRIL. *Immunity* 26 812–826 17570691 10.1016/j.immuni.2007.04.014

[B68] Heiser R. A. Snyder C. M. St Clair J. Wysocki L. J. (2011). Aborted germinal center reactions and B cell memory by follicular T cells specific for a B cell receptor V region peptide. *J. Immunol.* 187 212–221 21622866 10.4049/jimmunol.1002328 PMC3133611

[B69] Holmes G. K. Asquith P. Stokes P. L. Cooke W. T. (1973). Cellular infiltrate of jejunal biopsies in adult coeliac disease (ACD) in relation to gluten withdrawal. *Gut* 14 429 4716533

[B70] Holtmeier W. Hennemann A. Caspary W. F. (2000). IgA and IgM V(H) repertoires in human colon: evidence for clonally expanded B cells that are widely disseminated. *Gastroenterology* 119 1253–1266 11054383 10.1053/gast.2000.20219

[B71] Hooper L. V. Macpherson A. J. (2010). Immune adaptations that maintain homeostasis with the intestinal microbiota. *Nat. Rev. Immunol.* 10 159–169 20182457 10.1038/nri2710

[B72] Husby S. Koletzko S. Korponay-Szabo I. R. Mearin M. L. Phillips A. Shamir R. (2012). European Society for Pediatric Gastroenterology, Hepatology, and Nutrition guidelines for the diagnosis of coeliac disease. *J. Pediatr. Gastroenterol. Nutr.* 54 136–160 22197856 10.1097/MPG.0b013e31821a23d0

[B73] Jabri B. Sollid L. M. (2009). Tissue-mediated control of immunopathology in coeliac disease. *Nat. Rev. Immunol.* 9 858–870 19935805 10.1038/nri2670

[B74] Jiang H. Q. Thurnheer M. C. Zuercher A. W. Boiko N. V. Bos N. A. Cebra J. J. (2004). Interactions of commensal gut microbes with subsets of B- and T-cells in the murine host. *Vaccine* 22 805–811 15040931 10.1016/j.vaccine.2003.11.022

[B75] Johansen F. E. Braathen R. Brandtzaeg P. (2001). The J chain is essential for polymeric Ig receptor-mediated epithelial transport of IgA. *J. Immunol.* 167 5185–5192 11673531 10.4049/jimmunol.167.9.5185

[B76] Kau A. L. Ahern P. P. Griffin N. W. Goodman A. L. Gordon J. I. (2011). Human nutrition, the gut microbiome and the immune system. *Nature* 474 327–336 21677749 10.1038/nature10213 PMC3298082

[B77] Kaukinen K. Peraaho M. Collin P. Partanen J. Woolley N. Kaartinen T. (2005). Small-bowel mucosal transglutaminase 2-specific IgA deposits in coeliac disease without villous atrophy: a prospective and randomized clinical study. *Scand. J. Gastroenterol.* 40 564–572 16036509 10.1080/00365520510023422

[B78] Kawamoto S. Tran T. H. Maruya M. Suzuki K. Doi Y. Tsutsui Y. (2012). The inhibitory receptor PD-1 regulates IgA selection and bacterial composition in the gut. *Science* 336 485–489 22539724 10.1126/science.1217718

[B79] Kett K. Scott H. Fausa O. Brandtzaeg P. (1990). Secretory immunity in celiac disease: cellular expression of immunoglobulin A subclass and joining chain. *Gastroenterology* 99 386–392 2194895 10.1016/0016-5085(90)91020-7

[B80] Kilander A. F. Nilsson L. A. Gillberg R. (1987). Serum antibodies to gliadin in coeliac disease after gluten withdrawal. *Scand. J. Gastroenterol.* 22 29–34 3563409 10.3109/00365528708991852

[B81] Koninckx C. R. Giliams J. P. Polanco I. Pena A. S. (1984). IgA antigliadin antibodies in celiac and inflammatory bowel disease. *J. Pediatr. Gastroenterol. Nutr.* 3 676–682 6502368 10.1097/00005176-198411000-00006

[B82] Korponay-Szabo I. R. Halttunen T. Szalai Z. Laurila K. Kiraly R. Kovacs J. B. (2004). *In vivo* targeting of intestinal and extraintestinal transglutaminase 2 by coeliac autoantibodies. *Gut* 53 641–648 15082580 10.1136/gut.2003.024836 PMC1774023

[B83] Kroese F. G. Ammerlaan W. A. Deenen G. J. Adams S. Herzenberg L. A. Kantor A. B. (1995). A dual origin for IgA plasma cells in the murine small intestine. *Adv. Exp. Med. Biol.* 371A 435–440 8525961 10.1007/978-1-4615-1941-6_91

[B84] Labrooy J. T. Hohmann A. W. Davidson G. P. Hetzel P. A. Johnson R. B. Shearman D. J. (1986). Intestinal and serum antibody in coeliac disease: a comparison using ELISA. *Clin. Exp. Immunol.* 66 661–668 3568454 PMC1542459

[B85] Lancaster-Smith M. Kumar P. Marks R. Clark M. L. Dawson A. M. (1974). Jejunal mucosal immunoglobulin-containing cells and jejunal fluid immunoglobulins in adult coeliac disease and dermatitis herpetiformis. *Gut* 15 371–376 18668846 10.1136/gut.15.5.371 PMC1412914

[B86] Lanzavecchia A. (1985). Antigen-specific interaction between T and B cells. *Nature* 314 537–539 3157869 10.1038/314537a0

[B87] Lebreton C. Menard S. Abed J. Cruz-Moura I. Coppo R. Dugave C. (2012). Interactions among secretory immunogloblulin A, CD71, and transglutaminase-2 affect permeability of intestinal epithelial cells to gliadin peptides. *Gastroenterology* 143 698–707 22750506 10.1053/j.gastro.2012.05.051

[B88] Levesque M. C. Moody M. A. Hwang K. K. Marshall D. J. Whitesides J. F. Amos J. D. (2009). Polyclonal B cell differentiation and loss of gastrointestinal tract germinal centers in the earliest stages of HIV-1 infection. *PLoS Med.* 6 e1000107 10.1371/journal.pmed.1000107 PMC2702159 19582166

[B89] Lindner C. Wahl B. Fohse L. Suerbaum S. Macpherson A. J. Prinz I. (2012). Age, microbiota, and T cells shape diverse individual IgA repertoires in the intestine. *J. Exp. Med.* 209 365–377 22249449 10.1084/jem.20111980 PMC3280880

[B90] Litinskiy M. B. Nardelli B. Hilbert D. M. He B. Schaffer A. Casali P. (2002). DCs induce CD40-independent immunoglobulin class switching through BLyS and APRIL. *Nat. Immunol.* 3 822–829 12154359 10.1038/ni829 PMC4621779

[B91] Lorand L. Graham R. M. (2003). Transglutaminases: crosslinking enzymes with pleiotropic functions. *Nat. Rev. Mol. Cell Biol.* 4 140–156 12563291 10.1038/nrm1014

[B92] Lycke N. Kilander A. Nilsson L. A. Tarkowski A. Werner N. (1989). Production of antibodies to gliadin in intestinal mucosa of patients with coeliac disease: a study at the single cell level. *Gut* 30 72–77 2920930 10.1136/gut.30.1.72 PMC1378234

[B93] MacLennan I. C. Toellner K. M. Cunningham A. F. Serre K. Sze D. M. Zuniga E. (2003). Extrafollicular antibody responses. *Immunol. Rev.* 194 8–18 12846803 10.1034/j.1600-065x.2003.00058.x

[B94] Macpherson A. Khoo U. Y. Forgacs I. Philpott-Howard J. Bjarnason I. (1996). Mucosal antibodies in inflammatory bowel disease are directed against intestinal bacteria. *Gut* 38 365–375 8675088 10.1136/gut.38.3.365 PMC1383064

[B95] Macpherson A. J. Gatto D. Sainsbury E. Harriman G. R. Hengartner H. Zinkernagel R. M. (2000). A primitive T cell-independent mechanism of intestinal mucosal IgA responses to commensal bacteria. *Science* 288 2222–2226 10864873 10.1126/science.288.5474.2222

[B96] Maki M. Sulkanen S. Collin P. (1998). Antibodies in relation to gluten intake. *Dig. Dis.* 16 330–332 10207216 10.1159/000016885

[B97] Marietta E. V. Murray J. A. (2012). Animal models to study gluten sensitivity. *Semin. Immunopathol.* 34 497–511 22572887 10.1007/s00281-012-0315-y PMC3410984

[B98] Marshall J. L. Zhang Y. Pallan L. Hsu M. C. Khan M. Cunningham A. F. (2011). Early B blasts acquire a capacity for Ig class switch recombination that is lost as they become plasmablasts. *Eur. J. Immunol.* 41 3506–3512 21932446 10.1002/eji.201141762

[B99] Marzari R. Sblattero D. Florian F. Tongiorgi E. Not T. Tommasini A. (2001). Molecular dissection of the tissue transglutaminase autoantibody response in celiac disease. *J. Immunol.* 166 4170–4176 11238668 10.4049/jimmunol.166.6.4170

[B100] Matysiak-Budnik T. Moura I. C. Arcos-Fajardo M. Lebreton C. Menard S. Candalh C. (2008). Secretory IgA mediates retrotranscytosis of intact gliadin peptides via the transferrin receptor in celiac disease. *J. Exp. Med.* 205 143–154 18166587 10.1084/jem.20071204 PMC2234361

[B101] Mayer M. Greco L. Troncone R. Grimaldi M. Pansa G. (1989). Early prediction of relapse during gluten challenge in childhood celiac disease. *J. Pediatr. Gastroenterol. Nutr.* 8 474–479 2723938 10.1097/00005176-198905000-00009

[B102] Meresse B. Malamut G. Cerf-Bensussan N. (2012). Celiac disease: an immunological jigsaw. *Immunity* 36 907–919 22749351 10.1016/j.immuni.2012.06.006

[B103] Mesin L. Di Niro R. Thompson K. M. Lundin K. E. Sollid L. M. (2011). Long-lived plasma cells from human small intestine biopsies secrete immunoglobulins for many weeks *in vitro*. *J. Immunol.* 187 2867–2874 21841131 10.4049/jimmunol.1003181

[B104] Molberg O. McAdam S. N. Korner R. Quarsten H. Kristiansen C. Madsen L. (1998). Tissue transglutaminase selectively modifies gliadin peptides that are recognized by gut-derived T cells in celiac disease. *Nat. Med.* 4 713–717 9623982 10.1038/nm0698-713

[B105] Mombaerts P. Mizoguchi E. Ljunggren H. G. Iacomini J. Ishikawa H. Wang L. (1994). Peripheral lymphoid development and function in TCR mutant mice. *Int. Immunol.* 6 1061–1070 7947457 10.1093/intimm/6.7.1061

[B106] Mora J. R. Iwata M. Eksteen B. Song S. Y. Junt T. Senman B. (2006). Generation of gut-homing IgA-secreting B cells by intestinal dendritic cells. *Science* 314 1157–1160 17110582 10.1126/science.1132742

[B107] Muramatsu M. Kinoshita K. Fagarasan S. Yamada S. Shinkai Y. Honjo T. (2000). Class switch recombination and hypermutation require activation-induced cytidine deaminase (AID), a potential RNA editing enzyme. *Cell* 102 553–563 11007474 10.1016/s0092-8674(00)00078-7

[B108] Myrsky E. Kaukinen K. Syrjanen M. Korponay-Szabo I. R. Maki M. Lindfors K. (2008). Coeliac disease-specific autoantibodies targeted against transglutaminase 2 disturb angiogenesis. *Clin. Exp. Immunol.* 152 111–119 18279443 10.1111/j.1365-2249.2008.03600.x PMC2384074

[B109] Neutra M. R. (1999). M cells in antigen sampling in mucosal tissues. *Curr. Top. Microbiol. Immunol.* 236 17–32 9893353 10.1007/978-3-642-59951-4_2

[B110] Neutra M. R. Mantis N. J. Kraehenbuhl J. P. (2001). Collaboration of epithelial cells with organized mucosal lymphoid tissues. *Nat. Immunol.* 2 1004–1009 11685223 10.1038/ni1101-1004

[B111] Ogra P. L. Karzon D. T. (1969). Distribution of poliovirus antibody in serum, nasopharynx and alimentary tract following segmental immunization of lower alimentary tract with poliovaccine. *J. Immunol.* 102 1423–1430 4977607

[B112] Osman A. A. Richter T. Stern M. Mothes T. (1996). The IgA subclass distributions of endomysium and gliadin antibodies in human sera are different. *Clin. Chim. Acta* 255 145–152 8937757 10.1016/0009-8981(96)06401-7

[B113] Pabst O. Mowat A. M. (2012). Oral tolerance to food protein. *Mucosal Immunol.* 5 232–239 22318493 10.1038/mi.2012.4 PMC3328017

[B114] Paparo F. Petrone E. Tosco A. Maglio M. Borrelli M. Salvati V. M. (2005). Clinical, HLA, and small bowel immunohistochemical features of children with positive serum antiendomysium antibodies and architecturally normal small intestinal mucosa. *Am. J. Gastroenterol.* 100 2294–2298 16181383 10.1111/j.1572-0241.2005.41134.x

[B115] Pelletier N. McHeyzer-Williams L. J. Wong K. A. Urich E. Fazilleau N. McHeyzer-Williams M. G. (2010). Plasma cells negatively regulate the follicular helper T cell program. *Nat. Immunol.* 11 1110–1118 21037578 10.1038/ni.1954 PMC3058870

[B116] Radbruch A. Muehlinghaus G. Luger E. O. Inamine A. Smith K. G. Dorner T. (2006). Competence and competition: the challenge of becoming a long-lived plasma cell. *Nat. Rev. Immunol.* 6 741–750 16977339 10.1038/nri1886

[B117] Rauhavirta T. Qiao S. W. Jiang Z. Myrsky E. Loponen J. Korponay-Szabo I. R. (2011). Epithelial transport and deamidation of gliadin peptides: a role for coeliac disease patient immunoglobulin A. *Clin. Exp. Immunol.* 164 127–136 21235541 10.1111/j.1365-2249.2010.04317.x PMC3074225

[B118] Reynaud C. A. Garcia C. Hein W. R. Weill J. C. (1995). Hypermutation generating the sheep immunoglobulin repertoire is an antigen-independent process. *Cell* 80 115–125 7813007 10.1016/0092-8674(95)90456-5

[B119] Rimoldi M. Chieppa M. Salucci V. Avogadri F. Sonzogni A. Sampietro G. M. (2005). Intestinal immune homeostasis is regulated by the crosstalk between epithelial cells and dendritic cells. *Nat. Immunol.* 6 507–514 15821737 10.1038/ni1192

[B120] Salmi T. T. Collin P. Jarvinen O. Haimila K. Partanen J. Laurila K. (2006a). Immunoglobulin A autoantibodies against transglutaminase 2 in the small intestinal mucosa predict forthcoming coeliac disease. *Aliment. Pharmacol. Ther.* 24 541–552 16886921 10.1111/j.1365-2036.2006.02997.x

[B121] Salmi T. T. Collin P. Korponay-Szabo I. R. Laurila K. Partanen J. Huhtala H. (2006b). Endomysial antibody-negative coeliac disease: clinical characteristics and intestinal autoantibody deposits. *Gut* 55 1746–1753 16571636 10.1136/gut.2005.071514 PMC1856451

[B122] Sato A. Hashiguchi M. Toda E. Iwasaki A. Hachimura S. Kaminogawa S. (2003). CD11b+ Peyer’s patch dendritic cells secrete IL-6 and induce IgA secretion from naive B cells. *J. Immunol.* 171 3684–3690 14500666 10.4049/jimmunol.171.7.3684

[B123] Savilahti E. Viander M. Perkkio M. Vainio E. Kalimo K. Reunala T. (1983). IgA antigliadin antibodies: a marker of mucosal damage in childhood coeliac disease. *Lancet* 1 320–322 6130332 10.1016/s0140-6736(83)91627-6

[B124] Sblattero D. Florian F. Not T. Ventura A. Bradbury A. Marzari R. (2000). Analyzing the peripheral blood antibody repertoire of a celiac disease patient using phage antibody libraries. *Hum. Antibodies* 9 199–205 11341173

[B125] Scheid J. F. Mouquet H. Feldhahn N. Seaman M. S. Velinzon K. Pietzsch J. (2009). Broad diversity of neutralizing antibodies isolated from memory B cells in HIV-infected individuals. *Nature* 458 636–640 19287373 10.1038/nature07930

[B126] Schumann M. Richter J. F. Wedell I. Moos V. Zimmermann-Kordmann M. Schneider T. (2008). Mechanisms of epithelial translocation of the alpha(2)-gliadin-33mer in coeliac sprue. *Gut* 57 747–754 18305066 10.1136/gut.2007.136366

[B127] Scott H. Ek J. Baklien K. Brandtzaeg P. (1980). Immunoglobulin-producing cells in jejunal mucosa of children with coeliac disease on a gluten-free diet and after gluten challenge. *Scand. J. Gastroenterol.* 15 81–88 6988938 10.3109/00365528009181436

[B128] Shih T. A. Meffre E. Roederer M. Nussenzweig M. C. (2002). Role of BCR affinity in T cell dependent antibody responses *in vivo*. *Nat. Immunol.* 3 570–575 12021782 10.1038/ni803

[B129] Shikina T. Hiroi T. Iwatani K. Jang M. H. Fukuyama S. Tamura M. (2004). IgA class switch occurs in the organized nasopharynx- and gut-associated lymphoid tissue, but not in the diffuse lamina propria of airways and gut. *J. Immunol.* 172 6259–6264 15128814 10.4049/jimmunol.172.10.6259

[B130] Shiner M. Ballard J. (1972). Antigen-antibody reactions in jejunal mucosa in childhood coeliac disease after gluten challenge. *Lancet* 1 1202–1205 4113189 10.1016/s0140-6736(72)90924-5

[B131] Sollid L. M. (2002). Coeliac disease: dissecting a complex inflammatory disorder. *Nat. Rev. Immunol.* 2 647–655 12209133 10.1038/nri885

[B132] Sollid L. M. Molberg O. McAdam S. Lundin K. E. (1997). Autoantibodies in coeliac disease: tissue transglutaminase – guilt by association? *Gut* 41 851–852 9462222 10.1136/gut.41.6.851 PMC1891617

[B133] Sollid L. M. Qiao S. W. Anderson R. P. Gianfrani C. Koning F. (2012). Nomenclature and listing of celiac disease relevant gluten T-cell epitopes restricted by HLA-DQ molecules. *Immunogenetics* 64 455–460 22322673 10.1007/s00251-012-0599-z PMC3349865

[B134] Soltoft J. (1970). Immunoglobulin-containing cells in non-tropical sprue. *Clin. Exp. Immunol.* 6 413–420 4193742 PMC1712693

[B135] Spencer J. Klavinskis L. S. Fraser L. D. (2012). The human intestinal IgA response; burning questions. *Front. Immunol.* 3:108 10.3389/fimmu.2012.00108 PMC3349913 22593756

[B136] Stavnezer J. Kang J. (2009). The surprising discovery that TGF beta specifically induces the IgA class switch. *J. Immunol.* 182 5–7 19109126 10.4049/jimmunol.182.1.5

[B137] Stern M. Dietrich R. (1982). Gliadin- and immunoglobulin-containing cells of small intestinal lamina propria in childhood coeliac disease. *Eur. J. Pediatr.* 139 13–17 6756925 10.1007/BF00442071

[B138] Strugnell R. A. Wijburg O. L. (2010). The role of secretory antibodies in infection immunity. *Nat. Rev. Microbiol.* 8 656–667 20694027 10.1038/nrmicro2384

[B139] Su W. Gordon J. N. Barone F. Boursier L. Turnbull W. Mendis S. (2008). Lambda light chain revision in the human intestinal IgA response. *J. Immunol.* 181 1264–1271 18606680 10.4049/jimmunol.181.2.1264 PMC7116002

[B140] Sulkanen S. Halttunen T. Laurila K. Kolho K. L. Korponay-Szabo I. R. Sarnesto A. (1998). Tissue transglutaminase autoantibody enzyme-linked immunosorbent assay in detecting celiac disease. *Gastroenterology* 115 1322–1328 9834257 10.1016/s0016-5085(98)70008-3

[B141] Tezuka H. Abe Y. Iwata M. Takeuchi H. Ishikawa H. Matsushita M. (2007). Regulation of IgA production by naturally occurring TNF/iNOS-producing dendritic cells. *Nature* 448 929–933 17713535 10.1038/nature06033

[B142] Toellner K. M. Jenkinson W. E. Taylor D. R. Khan M. Sze D. M. Sansom D. M. (2002). Low-level hypermutation in T cell-independent germinal centers compared with high mutation rates associated with T cell-dependent germinal centers. *J. Exp. Med.* 195 383–389 11828014 10.1084/jem.20011112 PMC2193598

[B143] Tosco A. Maglio M. Paparo F. Rapacciuolo L. Sannino A. Miele E. (2008). Immunoglobulin A anti-tissue transglutaminase antibody deposits in the small intestinal mucosa of children with no villous atrophy. *J. Pediatr. Gastroenterol. Nutr.* 47 293–298 18728524 10.1097/MPG.0b013e3181677067

[B144] Toyama H. Okada S. Hatano M. Takahashi Y. Takeda N. Ichii H. (2002). Memory B cells without somatic hypermutation are generated from Bcl6-deficient B cells. *Immunity* 17 329–339 12354385 10.1016/s1074-7613(02)00387-4

[B145] Tursi A. Brandimarte G. Giorgetti G. M. (2003). Prevalence of antitissue transglutaminase antibodies in different degrees of intestinal damage in celiac disease. *J. Clin. Gastroenterol.* 36 219–221 12590232 10.1097/00004836-200303000-00007

[B146] Valletta E. A. Trevisiol D. Mastella G. (1990). IgA anti-gliadin antibodies in the monitoring of gluten challenge in celiac disease. *J. Pediatr. Gastroenterol. Nutr.* 10 169–173 2303968 10.1097/00005176-199002000-00004

[B147] van de Wal Y. Kooy Y. Van Veelen P. Pena S. Mearin L. Papadopoulos G. (1998). Selective deamidation by tissue transglutaminase strongly enhances gliadin-specific T cell reactivity. *J. Immunol.* 161 1585–1588 9712018

[B148] Victor K. D. Randen I. Thompson K. Forre O. Natvig J. B. Fu S. M. (1991). Rheumatoid factors isolated from patients with autoimmune disorders are derived from germline genes distinct from those encoding the Wa, Po, and Bla cross-reactive idiotypes. *J. Clin. Invest.* 87 1603–1613 2022732 10.1172/JCI115174 PMC295243

[B149] Victora G. D. Nussenzweig M. C. (2012). Germinal centers. *Annu. Rev. Immunol.* 30 429–457 22224772 10.1146/annurev-immunol-020711-075032

[B150] Wei M. Shinkura R. Doi Y. Maruya M. Fagarasan S. Honjo T. (2011). Mice carrying a knock-in mutation of Aicda resulting in a defect in somatic hypermutation have impaired gut homeostasis and compromised mucosal defense. *Nat. Immunol.* 12 264–270 21258321 10.1038/ni.1991

[B151] Weiner H. L. Da Cunha A. P. Quintana F. Wu H. (2011). Oral tolerance. *Immunol. Rev.* 241 241–259 21488901 10.1111/j.1600-065X.2011.01017.x PMC3296283

[B152] Weitkamp J. H. Kallewaard N. Kusuhara K. Bures E. Williams J. V. Lafleur B. (2003). Infant and adult human B cell responses to rotavirus share common immunodominant variable gene repertoires. *J. Immunol.* 171 4680–4688 14568943 10.4049/jimmunol.171.9.4680

[B153] William J. Euler C. Christensen S. Shlomchik M. J. (2002). Evolution of autoantibody responses via somatic hypermutation outside of germinal centers. *Science* 297 2066–2070 12242446 10.1126/science.1073924

[B154] Wood G. M. Howdle P. D. Trejdosiewicz L. K. Losowsky M. S. (1987). Jejunal plasma cells and *in vitro* immunoglobulin production in adult coeliac disease. *Clin. Exp. Immunol.* 69 123–132 3652527 PMC1542244

[B155] Wrammert J. Smith K. Miller J. Langley W. A. Kokko K. Larsen C. (2008). Rapid cloning of high-affinity human monoclonal antibodies against influenza virus. *Nature* 453 667–671 18449194 10.1038/nature06890 PMC2515609

[B156] Yuvaraj S. Dijkstra G. Burgerhof J. G. Dammers P. M. Stoel M. Visser A. (2009). Evidence for local expansion of IgA plasma cell precursors in human ileum. *J. Immunol.* 183 4871–4878 19786537 10.4049/jimmunol.0901315

